# Pharmacometrics-Based Considerations for the Design of a Pharmacogenomic Clinical Trial Assessing Irinotecan Safety

**DOI:** 10.1007/s11095-021-03024-w

**Published:** 2021-03-17

**Authors:** Iris K. Minichmayr, Mats O. Karlsson, Siv Jönsson

**Affiliations:** Department of Pharmacy, Uppsala University, Box 580, 75123 Uppsala, Sweden

**Keywords:** irinotecan model, neutropenia, pharmacogenomics, study design, UGT1A1

## Abstract

**Purpose:**

Pharmacometric models provide useful tools to aid the rational design of clinical trials. This study evaluates study design-, drug-, and patient-related features as well as analysis methods for their influence on the power to demonstrate a benefit of pharmacogenomics (PGx)-based dosing regarding myelotoxicity.

**Methods:**

Two pharmacokinetic and one myelosuppression model were assembled to predict concentrations of irinotecan and its metabolite SN-38 given different UGT1A1 genotypes (poor metabolizers: CL_SN-38_: -36%) and neutropenia following conventional versus PGx-based dosing (350 versus 245 mg/m^2^ (-30%)). Study power was assessed given diverse scenarios (*n* = 50–400 patients/arm, parallel/crossover, varying magnitude of CL_SN-38_, exposure-response relationship, inter-individual variability) and using model-based data analysis versus conventional statistical testing.

**Results:**

The magnitude of CL_SN-38_ reduction in poor metabolizers and the myelosuppressive potency of SN-38 markedly influenced the power to show a difference in grade 4 neutropenia (<0.5·10^9^ cells/L) after PGx-based versus standard dosing. To achieve >80% power with traditional statistical analysis (χ^2^/McNemar’s test, α = 0.05), 220/100 patients per treatment arm/sequence (parallel/crossover study) were required. The model-based analysis resulted in considerably smaller total sample sizes (*n* = 100/15 given parallel/crossover design) to obtain the same statistical power.

**Conclusions:**

The presented findings may help to avoid unfeasible trials and to rationalize the design of pharmacogenetic studies.

**Supplementary Information:**

The online version contains supplementary material available at 10.1007/s11095-021-03024-w.

## Introduction

The accumulating knowledge of genomic variations between patients and their association with adverse drug reactions has prompted an emerging model of personalized medicine involving genotype-informed treatment stratification (1). With the ultimate goal to improve patient outcome and therapy safety, pharmacogenomics (PGx)-based treatment recommendations have been developed inter alia by the Clinical Pharmacogenetics Implementation Consortium and the Dutch Pharmacogenetics Working Group (DPWG) (2–4). However, their widespread adoption in routine clinical practice has been impeded by several challenges, including the lack of evidence demonstrating their clinical utility (5). To fill this gap and evaluate a potential positive impact of PGx-based dosing, clinical trials are required.

The design of pharmacogenetic trials is critical for their efficiency and success, though not trivial. Key considerations include the choice of a relevant study endpoint, the expected effect size (e.g. the magnitude of reduction of adverse drug reactions after PGx-based versus conventional dosing), and the sample size associated therewith. The number of subjects should be sufficiently large to detect the defined effect size with reasonable power, though at the same time be feasible with respect to the allotted budget and time. As a further complication, the availability of potentially recruitable patients might be limited depending on the studied genetically defined patient (sub-)population.

Pharmacometric models and simulations provide useful tools to aid the rational design of clinical trials. Model-based approaches have rendered growing contribution to inform decision-making processes in drug development, not least because regulatory agencies have increasingly advocated their application (6), but also in the clinical setting to support dosing recommendations, improve therapeutic efficacy and safety (7). Pharmacokinetic/pharmacodynamic (PK/PD) models allow to explore the consequences of dosing on drug concentrations and on favorable or adverse effects and enable realistic simulations of clinical trials and their power, with the goal to maximize study informativeness and efficiency.

Model-based data analysis of clinical trials entails the advantage of simultaneously analyzing and thus making use of all available data of the investigated subjects (typically repeated measurements over time) and has the potential to increase the overall information content of clinical trials compared to traditional statistical analysis methods. In contrast, the latter non-model-based approaches conventionally only consider one single time point of the concentration- or effect-time profile (8). The utility of clinical trial simulations and model-based data analysis methods to inform the design of clinical studies has been demonstrated across various therapeutic fields (9–11). In pharmacogenomics, pharmacometric models have foremost been used to enhance the understanding of the impact of genetic variants on drug exposure and various outcomes rather than for clinical trial design. For example, irinotecan among other chemotherapeutic drugs has been the subject of different pharmacogenomic investigations, although no combined PK/PD model also illustrating the impact of PGx has been published yet (12).

The topoisomerase I inhibitor irinotecan (CPT-11, Camptosar®) constitutes a valuable treatment option for colorectal cancer and other solid tumors. It is approved as single-agent regimen at a dose of 350 mg/m^2^ (administered as i.v. infusion over 90 min every 3 weeks) and at lower doses as part of combination therapy regimens (13). Irinotecan (t_1/2_CPT-11_ = 6–12 h) is bioactivated by hydrolysis into its 100–1000 times more potent metabolite SN-38 (t_1/2_SN-38_ = 10–20 h) and further metabolized by the enzyme uridine diphosphate-glucuronosyltransferase 1A1 (UGT1A1) to the inactive SN-38-glucuronide (SN-38-G) (13–15). Aberrant UGT1A1 genetic variants may confer reduced UGT1A1 activity, as shown by a population pharmacokinetic analysis revealing a 36% reduced clearance of the metabolite SN-38 in patients with genotypes *28/*28 and *1/*28 (in the following referred to as “poor metabolizers”) compared to patients with UGT1A1*1/*1 (“wild-type”) genotype (16, 17). Particularly patients homozygous for the UGT1A*28 allele display a dose-dependent increased risk of irinotecan-induced grade 3–4 hematologic toxicity (14, 18, 19). Thus, a PGx-based reduction of the initial dose by 30% for standard doses >250 mg/m^2^ has been recommended for poor metabolizers (*28/*28) (3).

The objective of the current study was to evaluate the impact of varying study design-, drug- and patient-related features on the statistical power to discern a significant difference in the occurrence of adverse events after PGx-based versus conventional dosing. Furthermore, a pharmacometric model-based approach of data analysis was illustrated in comparison to traditional statistical testing with respect to study power. The objectives are exemplified by a population PK/PD model for irinotecan integrating the impact of PGx on drug exposure and toxicity response.

## Methods

Several pharmacometric model sources were integrated to enable the simulation of irinotecan and metabolite concentrations for different genetic variants of UGT1A1 and an associated response reflected by the change of neutrophils during chemotherapy. The combined PK/PD model served as the basis to evaluate alternative study designs and data analysis methods for their power to show a benefit of PGx-based versus standard dosing of irinotecan with respect to the occurrence of severe neutropenia.

### Population Models

#### Population Pharmacokinetic Model for Irinotecan, SN-38 and SN-38G

**Fig. 1:**
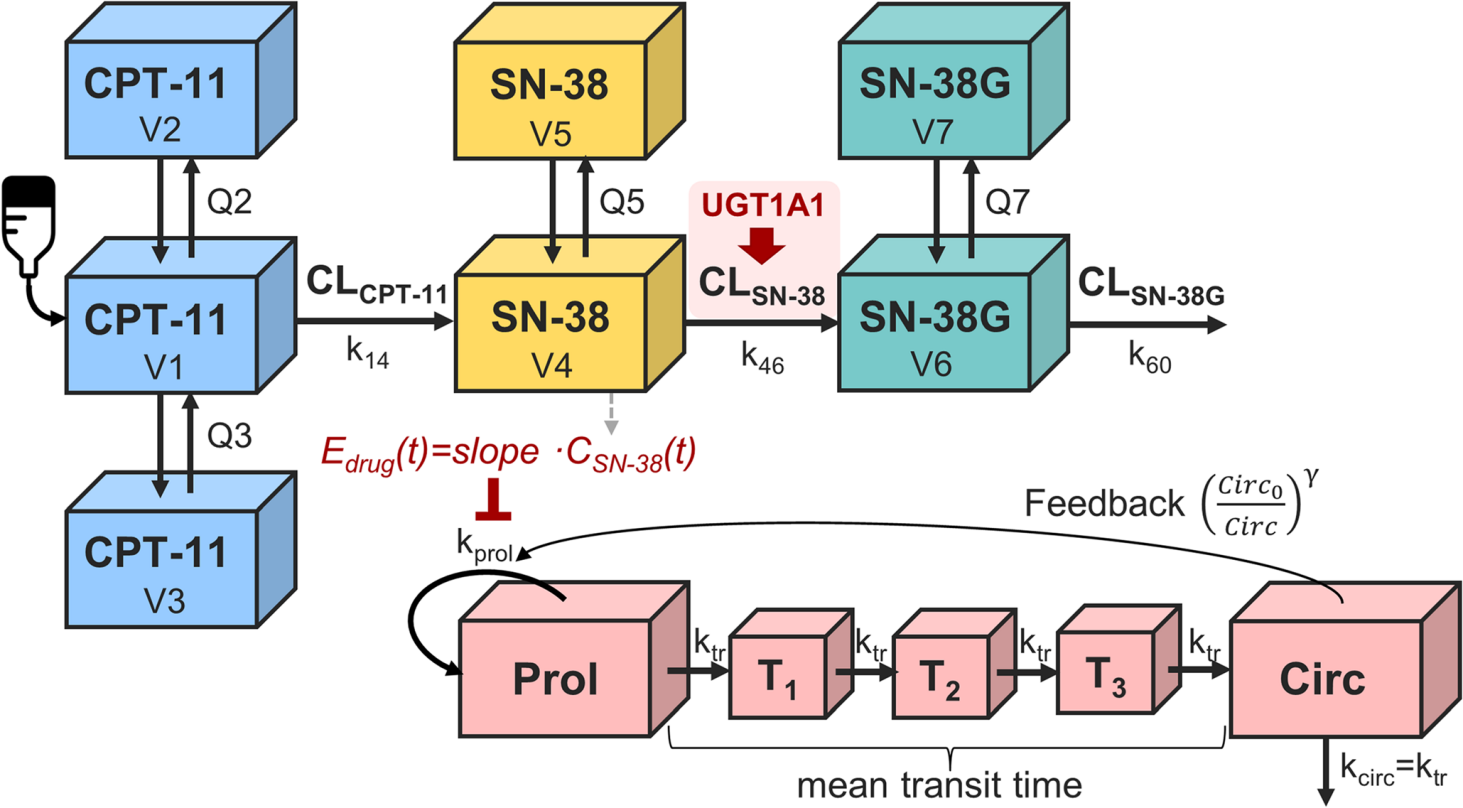
Integrated PK/PD model linking irinotecan dosing to neutropenia as an important adverse drug reaction. CPT-11: irinotecan; SN-38: 7-ethyl-10-hydroxycamptothecin; SN-38G: SN-38 glucuronide. UGT: uridine diphosphate glucuronosyl-transferase; CL: clearance; Q: intercompartmental CL; k: rate constants; SN-38 and SN-38G were assumed to be formed from CPT-11 and SN-38 according to first-order processes. The semi-mechanistic model of myelosuppression allowed to describes the time course of neutrophils: it consists of a proliferating compartment (Prol) representing stem and progenitor cells sensitive to the myelosuppressive agent, a compartment of circulating neutrophils in blood (Circ; Circ_0_: baseline value), and a maturation chain with three transit compartments (T1-T3), enabling the prediction of a time delay between drug administration and the observed myelosuppressive effect. γ: feedback parameter, accounting for the impact of endogenous growth factors on the proliferation rate; k_prol_: proliferation rate constant determining the rate of cell division.

A previously published multi-compartmental PK model was used as a starting point to simultaneously describe plasma concentrations of CPT-11 (number of individuals n_ID_ = 109), SN-38 (n_ID_ = 109) and SN-38G (n_ID_ = 83) in patients with solid tumors receiving 300 mg/m^2^ (median; range: 100–350 mg/m^2^) irinotecan as i.v. infusions over 1.5 h (range 0.75–2.25 h) every three weeks (20). The model was extended with a covariate effect quantifying the impact of patients’ UGT1A1 genotype on the elimination of the active metabolite SN-38 (CL_SN-38_ -35.7% for UGT1A1*28/*28 or*1/*28; see Fig. 1) (17). The original model accommodating the PGx-CL_SN-38_ relationship was built upon data from 72 Caucasian patients with solid tumors receiving irinotecan infusions (65–350 mg/m^2^, 60–90 min) every two weeks (17, 20). A mixture modeling approach was used, assuming two subpopulations (patients with wild-type UGT1A1 genotype and poor metabolizers with CL_SN-38_ -35.7%) with separately estimated CL_SN-38_ and intercompartmental clearance (Q_SN-38_), when updating model parameters given the extended clearance model. The proportion of patients with UGT1A1*28/*28 and *1/*28 was fixed to 62% according to the prevalence of these genotypes in Caucasians (21). Body surface area (BSA) was added as a covariate on the distribution and elimination parameters of all three entities CPT-11, SN-38 and SN-38G.

#### Semi-Mechanistic PK/PD Model of Neutropenia

Neutropenia during irinotecan therapy was described using a semi-mechanistic PK/PD model of chemotherapy-induced myelosuppression (22). The model was based on 20 patients with solid tumors, who contributed 79 observations following single-agent irinotecan therapy and constituted a subset of the population underlying the PK model for irinotecan (20, 22). The myelosuppression model mimics the maturation of blood cells and distinguishes between *drug-specific* parameters and three *system-related* parameters (*baseline*, *mean transit time* and *feedback,* see Fig. 1). The model was adapted with respect to the *drug-specific* parameters such that the active metabolite SN-38 acted as the driving force inducing myelosuppression. The effect of SN-38 concentrations on the proliferation of sensitive cells was described by an inhibitory linear model and quantified by a *slope* parameter (drug effect *E*_*drug*_*(t) = slope · conc*).

#### Evaluation of the Population PK/PD Models

Model evaluation comprised standard goodness-of-fit plots (e.g. observed versus population and individual predicted concentrations, conditional weighted residuals versus population predicted concentrations and time), precision of parameter estimates and scientific plausibility. Model comparison was further guided by statistical significance, i.e. the difference in the objective function value (i.e. approximate −2 log likelihood) between nested models (ΔOFV >3.84, α = 0.05, *df* = 1). Visual predictive checks (VPC), i.e. assessment of 1000 concentration-time profiles simulated based on the final parameter estimates, were used to evaluate the predictive performance of the model.

### Study Design Evaluation to Assess the Power to Show a Clinical Benefit of PGx-Based Dosing

**Fig. 2:**
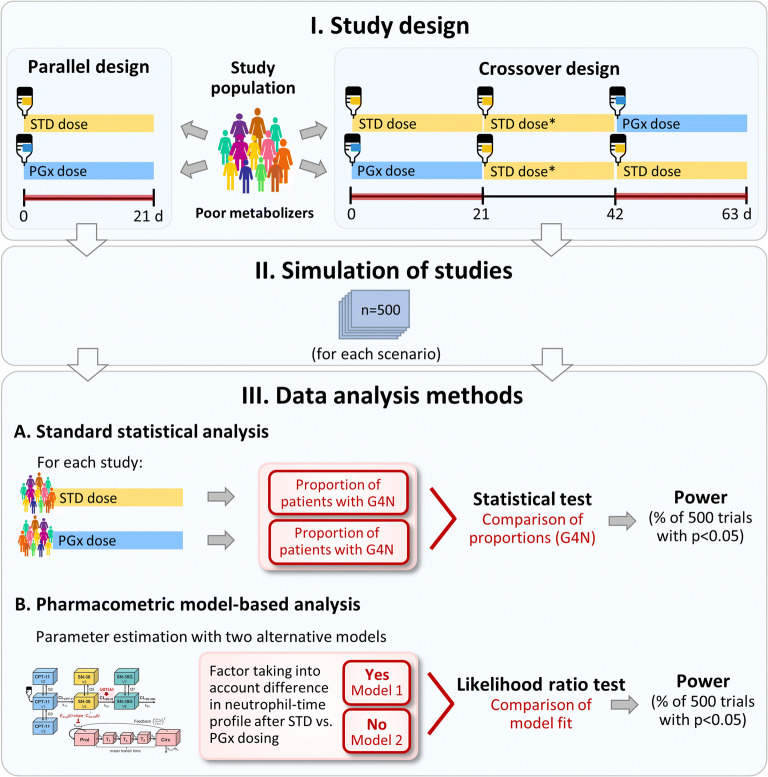
Workflow of the study design evaluation. Upper panel: The study population of poor metabolizers was randomized into two treatment arms (parallel study design) or two treatment sequences (crossover study design). A 3-period/2-sequence/2-treatment crossover design was chosen to ensure that the dose preceding the assessment of grade 4 neutropenia was the same for both treatment sequences. *For clinical study design evaluations, an alternative study design with PGx-based dosing instead of standard dosing as the second study period was additionally investigated. Red bars mark study periods for which myelosuppression was determined in the standard statistical analysis. Middle panel: 500 studies were simulated given a specific study design and scenario. Lower panel: Workflow of (A) standard statistical analysis and (B) pharmacometric model-based analysis to determine a potential difference in neutropenia after standard versus PGx-based dosing. STD: standard; PGx: pharmacogenomics-based; G4N: grade 4 neutropenia.

The combined population PK/PD model was used for simulations (*n* = 500–1000) to illustrate new, yet unstudied “what-if” scenarios, including the consequences of UGT1A1 genotype and dosing on drug exposure and myelosuppression as a relevant clinical toxicity endpoint. Furthermore, we systematically investigated reasonable study conditions including the impact of varying study design features, drug-, patient- and model-related aspects as well as methods of data analysis on the statistical power of a clinical trial aiming to observe a significant change in neutropenia after PGx-based versus conventional dosing in poor metabolizers (Fig. 2). First, a *default scenario* with respect to study design, patient population and features of the model was set up. Furthermore, *alternative study designs* and model aspects of importance were systematically varied and explored for their impact on study power.

For each specific investigated study design, 500 replicates of a clinical trial were generated by stochastic simulations with the PK/PD model, considering PK and PD inter-individual and residual variability. Simulated baseline concentrations of neutrophils were constrained to a clinically realistic lower limit of 1.5·10^9^ cells/L, which was in agreement with the recommendation not to start irinotecan therapy if values are lower, as well as with patient data underlying the two combined population PK models (13, 17, 20). The upper baseline limit for neutrophils was set to 15·10^9^ cells/L.

#### Default Clinical Trial Design Scenario

A virtual *study population* of poor metabolizers was created. BSA was sampled from a normal distribution (mean 1.87 m^2^, standard deviation 0.25 m^2^) truncated at 1.3–2.5 m^2^. The selected range of BSA reflected the cancer patient population underlying the used population PK model for irinotecan (20) and results of a vast multicenter study on the BSA area of adult cancer patients (23). To assess myelotoxicity, a clinically feasible *sampling scheme* for neutrophils with weekly intervals (days 0–7–14-21) was chosen. The observation period of three weeks was in accordance with the dosing interval of the approved single-agent therapy regimen (13).

A parallel *study design* was chosen as the default scenario (see Fig. 2). A *sample size* of *n* = 200 patients per treatment arm was selected after a preliminary exploratory statistical analysis (see Supplementary Material). Patients were randomized to receive irinotecan either as standard or PGx-based *dosing regimen*: The single-agent *standard dosing* regimen of 350 mg/m^2^ irinotecan was selected according to the product label (13) and in agreement with the mean dosing scheme underlying the original semi-mechanistic PK/PD model for myelosuppression (22). The dose of 350 mg/m^2^ was within the range for which PGx-based dose adjustment is recommended (>250 mg/m^2^ (3)). The *PGx-based dosing* regimen (245 mg/m^2^ for patients with *28/*28 genotype) was chosen according to the recommendation of the DPWG guideline to reduce the initial standard dose by 30% (3). For all doses, single-dose i.v. infusion over 90 min was assumed (13).

#### Alternative Clinical Trial Scenarios

The *study population* of the alternative studies and *sampling scheme* for neutrophils corresponded to those of the default scenario. The investigated alternative studies deviated from the default scenario with respect to one or more study design features, drug-specific characteristics and model parameters as follows: Apart from parallel *study design*, studies with (3-period/2-sequence/2-treatment) crossover design were simulated (see Fig. 2). In the trial simulations, the *sample size* was varied from *n* = 50–400 patients per treatment group (parallel design) or sequence (crossover design). Myelotoxicity was assessed after the first dose, and in the cross-over design also after the third dose.

Apart from the single-agent labelled standard dosing regimen and the PGx-based dosing regimen recommended in the DPWG guideline (see above), reduced *dosing regimens* as used in chemotherapeutic combination therapy regimens like mFolfirinox were included in the clinical trial evaluation (24) as follows. A reduced standard dose of 180 mg/m^2^ has previously been recommended for patients with UGT1A1*1/*1, together with further reduced relative *doses* of 50% and 75% for poor and intermediate metabolizers, i.e. only 90 mg/m^2^ for patients with UGT1A1*28/*28 and 135 mg/m^2^ for patients with UGT1A1*1/*28 (24). Single-dose administration (90 min i.v. infusion) was assumed for parallel study designs and a dosing interval of 21 days was applied in crossover study designs (13).

Different *aspects of the PK/PD model,* i.e. *magnitudes of parameters,* were investigated for their consequences on study power based on the default scenario: First, the *drug-related* potency parameter (slope = 26.8 μM^−1^) was varied from −50% to 400% of the original estimate in the myelosuppression model. Furthermore, the pharmacogenomic effect of the UGT1A1 genotype–reflected by the magnitude of CL_SN-38_ reduction in poor metabolizers and thus a *patient-related* model parameter–was tweaked and assessed for its impact on statistical power. Last, data were simulated with varying magnitudes of the unexplained inter-individual variability (IIV) terms associated with the two abovementioned parameters *slope* and *CL*_*SN-38*_ in order to evaluate the impact of variability on study power.

#### Data Analysis Methods

A *standard statistical* and a *pharmacometric model-based* approach were employed and compared to investigate the impact of design factors on the power to identify a significant change in myelotoxicity following PGx-based dosing (Fig. 2). Using *standard statistical analysis,* each of the simulated studies (*n* = 500) given a specific scenario was subjected to a statistical test assessing the difference in the *proportions of patients experiencing grade 4 neutropenia* (<0.5·10^9^ cells/L according to the Common Terminology Criteria for Adverse Events criteria (25)) after standard versus PGx-based dosing. Thus, myelotoxicity as the clinical endpoint was considered as the presence or absence of grade 4 neutropenia during treatment irrespective of the time of its occurrence and the magnitude of neutrophil reduction. Two-sided statistical tests with continuity correction (parallel design: χ^2^ test, crossover design: McNemar’s test) were applied. Study power was assessed at a significance level of 5% and corresponded to the percentage of studies for which a statistically significant difference in grade 4 neutropenia could be demonstrated after PGx-based versus standard dosing. Standard statistical analysis constituted the method of choice for the analysis of the alternative clinical trial scenarios.

The *pharmacometric model-based approach* investigated differences in myelotoxicity using SSE (stochastic simulation and estimation) for parallel design (default scenario) and crossover design (26). First, 500 datasets were simulated based on the same model and dataset features (e.g. dosing, sampling time) as in the standard statistical analysis. The PK/PD model was subsequently used to *estimate* model parameters based on the *entire time course of neutrophil concentrations* specified in the dataset, i.e. not only the time point of occurring grade 4 neutropenia. Two models were fitted to the simulated datasets: In Model 1 (‘full model’), a factor taking into account a potential difference in the neutrophil concentration-time profile after standard versus PGx-based dosing (representing a fraction of the standard dose) was estimated, whereas Model 2 (‘reduced model’) assumed no difference between the neutrophil-time courses after different dosing regimens. In both models, the drug-specific parameters *slope*, the corresponding *inter-individual variability* (ω_slope_) and the *residual variability* associated with neutrophil counts were estimated; all other (e.g. PK and system-related PD) parameters were fixed to values of the final PK/PD model. The performance of Models 1 and 2 was compared based on their parameter estimates and the likelihood ratio test. The percentage of studies showing a statistically significant difference in the fit of the models (*p* = 0.05, ΔOFV 3.84, *df* = 1) was determined, corresponding to the power to show a difference in the time course of myelotoxicity after PGx-based dose reduction. To visualize results, full power versus sample size curves were created for each SSE based on a parametric power estimation (PPE) algorithm facilitated with PsN, enabling the extrapolation of statistical power from one specific sample size to sample sizes beyond the originally simulated size (parallel design: *n* = 200/treatment arm; crossover design: *n* = 100/sequence) (27).

Nonlinear mixed-effects modeling and simulations were performed using NONMEM 7.3-7.4 (28) (ICON Clinical Research LLC, Gaithersburg, MD) and the first-order conditional estimation method (with interaction), assisted by PsN 4.7.0 (26) (Uppsala University, Sweden; https://uupharmacometrics.github.io/PsN). Statistical and graphical analyses were conducted using R 3.0.2 (R Core Team 2019. R Foundation for Statistical Computing, Vienna, Austria. https://www.R-project.org).

## Results

### Population Pharmacokinetic/Pharmacodynamic Model

The assembled population PK/PD model allowed to simultaneously predict the time courses of (i) plasma concentrations of irinotecan and its metabolites as well as (ii) neutrophils in patients with UGT1A1 wild-type and poor metabolizers. It integrated a UGT1A1-dependent reduction of the elimination of SN-38 (CL_SN-38_), resulting in higher SN-38 concentrations in poor metabolizers (genotypes *28/*28 and *1/*28; see supplementary Fig. S1). The PK/PD model of chemotherapy-induced myelosuppression adequately predicted neutropenia driven by the active metabolite SN-38, as demonstrated by a visual predictive check (see supplementary Fig. S2), and all parameters of the structural model were estimated with high certainty (relative standard error ≤ 28%). Inter-individual variability was implemented on all PK parameters and on the PD parameters *baseline, mean transit time* and *slope*. Either combined additive/proportional or additive residual unexplained variability models were used for each observed measurement entity (CPT-11, SN-38, SN-38G and neutrophils). The additive component of the residual variability for neutrophils was fixed to a value previously determined in a model based on the same dataset assessing the effect of CTP-11 on neutrophil concentrations. Table I presents the parameter estimates of the combined PK/PD model.

**Table I:** Parameter estimates of the combined pharmacokinetic/pharmacodynamic model for myelosuppression induced by irinotecan therapy

Parameter	Estimate	Parameter	Estimate
Structural model		Inter-individual variability	
*Pharmacokinetic component*		ω_CL_CPT-11_ [CV%]	32.6
CL_CPT-11_ [L/h]	31.6	ω_Q2_ [CV%]	86.1
Q2 [L/h]	114	ω_Q3_ [CV%]	49.5
Q3 [L/h]	9.89	ω_V1_	see Scal_ω_CLCPT11-V1_
V1 [L]	68.6	ω_V2_	see Scal_ω_CLCPT11-V2_
V2 [L]	67.2	ω_V3_	see Scal_ω_Q3CPT11-V3_
V3 [L]	127	ω_CL_SN-38_WT_ [CV%]	71.5
Scal_ω_CLCPT11-V1_	0.471	ω_CL_SN-38_PM_ [CV%]	37.3
Scal_ω_CLCPT11-V2_	0.496	ω_Q5_WT_ [CV%]	see Scal_ω_ CLSN38WT_Q5_
Scal_ω_Q3CPT11-V3_	0.893	ω_Q5_PM_ [CV%]	see Scal_ω_CLSN38PM_Q5_
CL_SN-38_WT_ [L/h]	1040	ω_V4_ [CV%]	105.2
CL_SN-38_PM_ [L/h; calc.]	(668.7)	ω_V5_ [CV%]	64.0
Q5_WT_ [L/h]	1690	ω_CL_SN-38G_ [CV%]	94.6
Q5_PM_ [L/h]	1290	ρ_CLSN38G-V6_ [%]	87.4
V4 [L]	411	ω_V6_ [CV%]	90.2
V5 [L]	60,000	ω_Q7_ [CV%]	93.0
Scal_ω_ CLSN38WT_Q5_	0.469	ω_V7_ [CV%]	71.1
Scal_ω_CLSN38PM_Q5_	1.62	ω_CIRC0_ [CV%]	29.2
CL_SN-38G_ [L/h]	77	ω_MTT_ [CV%]	21.9
V6 [L]	52.1	ω_SLO_ [CV%]	37.4
Q7 [L/h]	18	Residual variability	
V7 [L]	60.2	σ_NEU,add._ [·10^9^/L]	0.434
		σ_NEU,prop._ [CV%]	35.5
*Pharmacodymamic component*		σ_CPT-11,add._ [ng/mL]	1.3
Circ_0_ [·10^9^/L]	5.58	σ_CPT-11,prop._ [CV%]	16.7
MTT [h]	92.8	σ_SN-38,add._ [ng/mL]	0.484
γ	0.165	σ_SN-38,prop._ [CV%]	26.9
SLO [μM^−1^]	26.8	σ_SN-38G,add._ [ng/mL]	33.3

CPT-11: irinotecan; SN-38: 7-ethyl-10-hydroxycamptothecin; SN-38G: SN-38 glucuronide; CL: clearance; Q: intercompartmental CL; V: volume of distribution; Scal: scaling parameter for inter-individual variability (ratio of the standard deviations of the distributions for the two stated parameters, e.g. Scal_ω_CLCPT11-V1_ = ω_V1_/ω_CLCPT11_); WT: wild-type; PM: poor metabolizers; Circ_0_: baseline value of circulating cells; MTT: mean transit time; γ: feedback parameter; SLO: slope parameter quantifying the inhibitory drug effect on neutrophils; ω: parameter quantifying inter-individual variability associated with a structural parameter; %CV: coefficient of variation; ρ: correlation between inter-individual random effects; σ: parameter quantifying residual unexplained variability; NEU: neutrophils; add: additive; prop: proportional; parameter specifications are stated as outlined in Fig. 1. The relative standard errors were ≤ 28% for structural parameters and ≤ 35% (apart from IIV_MTT: 46%; Scal_ω_ CLSN38WT_Q5_: 84%) for random effects parameters. Distribution and elimination parameters of all three entities CPT-11, SN-38 and SN-38G were based on a body surface area of 1.87 m^2^

### Simulations to Illustrate the Consequences of UGT1A1 Genotype on Neutropenia

**Fig. 3:**
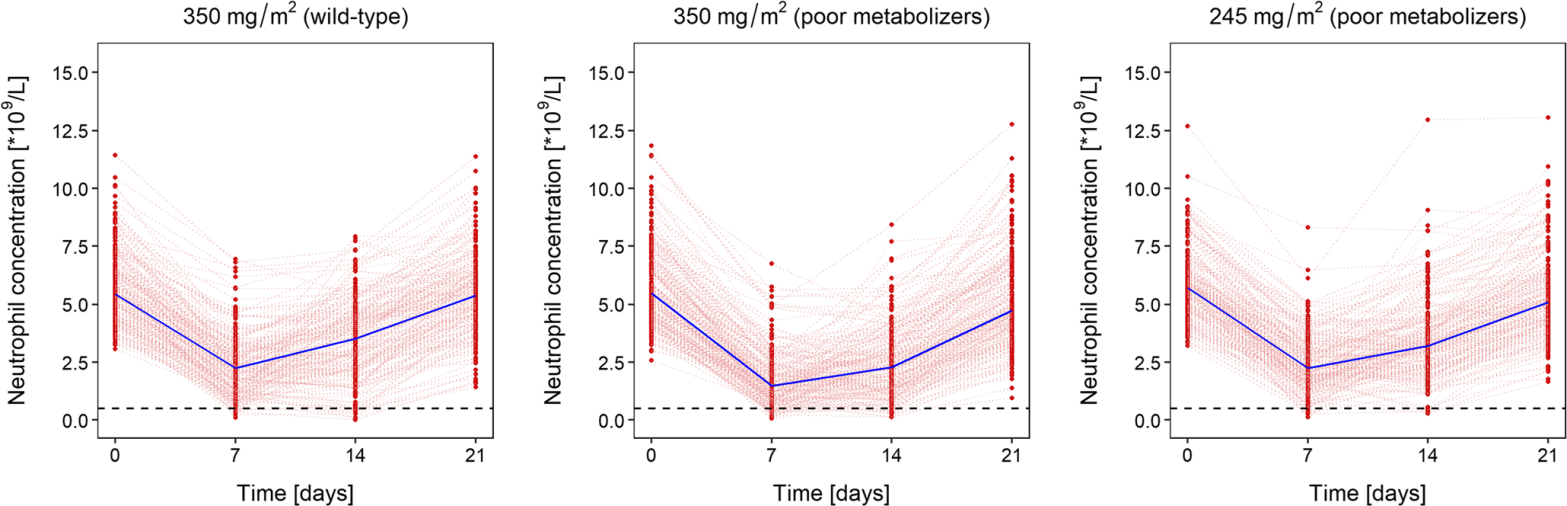
Time course of neutrophil concentrations in patients with genotype *1/*1 (wild-type, left panel) or *28/*28 and *1/*28 (“poor metabolizers”, middle and right panel). For wild-type UGT1A1, a standard dose of irinotecan (350 mg/m^2^) was simulated. For poor metabolizers, myelosuppression after a standard dose (350 mg/m^2^, middle panel) versus an individualized PGx-based dose (245 mg/m^2^, right panel) is shown. The presented exemplary stochastic simulations were based on 200 patients and considered inter-individual pharmacokinetic and pharmacodynamic variability in myelosuppression. The nadir occurs at different time points. The dashed horizontal line indicates grade 4 neutropenia (0.5·10^9^ cells/L).

Simulations enabled to show how differences in UGT1A1 genotype and consequently SN-38 exposure translated into differences in irinotecan-induced myelosuppression. The model predicted less pronounced neutropenia in patients with wild-type genotype compared to the UGT1A1*28 allele following the approved single-agent dose of irinotecan (350 mg/m^2^, Fig. 3). 13.6% of patients (*n* = 1000) with wild-type genotype (median; 95% prediction interval CI_95_ = 11.4–15.7%) and 20.5% of poor metabolizers (CI_95_ = 18.1–22.9%) displayed grade 4 neutropenia. PGx-based dosing reduced this proportion to 10.7% (CI_95_ = 8.6–12.5%), which corresponded to a relative reduction of 48% in the proportion and was comparable to patients with wild-type genotype after standard dosing. Given this decrease, the dose differences appeared to be large enough and the investigated model scenario sensitive enough regarding the clinical endpoint ‘grade 4 neutropenia’ and appropriate to elucidate diverse study design effects.

### Evaluation of Study Designs by Standard Statistical Analysis

**Fig. 4:**
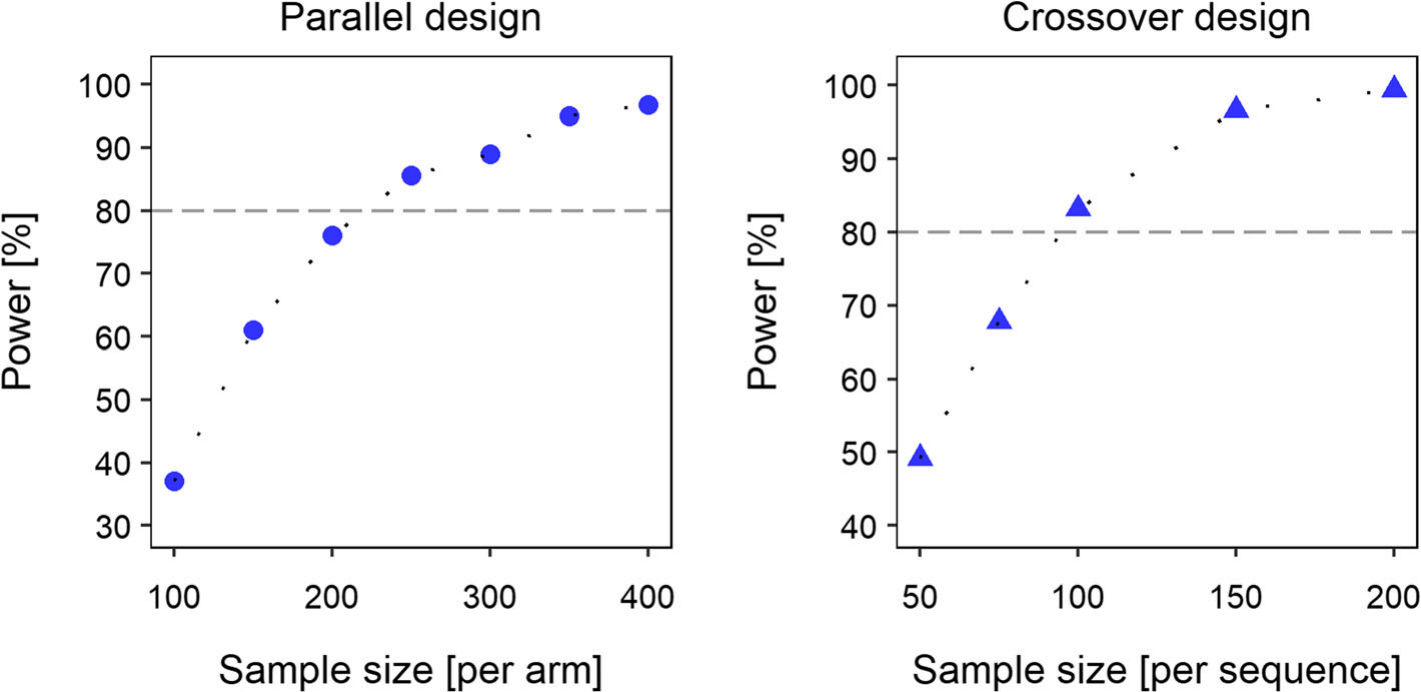
Impact of different study designs and varied sample sizes on study power. Left panel: parallel design; right panel: crossover design (see Fig. 2).

In a study with *parallel design*, ~220 patients per treatment arm would be required to show a significant difference between the proportions of patients displaying grade 4 neutropenia after PGx-based versus standard dosing with a power of >80% (see Fig. 4). When choosing a 3-period/2-sequence/2-treatment *crossover design*, the sample size of a study was reduced to <100 patients per treatment sequence given the same power. Similar results (with a deviation in power ≤ 3.66%) were obtained when choosing a modified crossover design with PGx-based dosing rather than standard dosing as the second study period.

Apart from sample size, the impact of further aspects related to study design or the PK/PD model on study power was explored using a parallel study with *n* = 200 patients per treatment arm.

#### Dosing Regimen

**Fig. 5:**
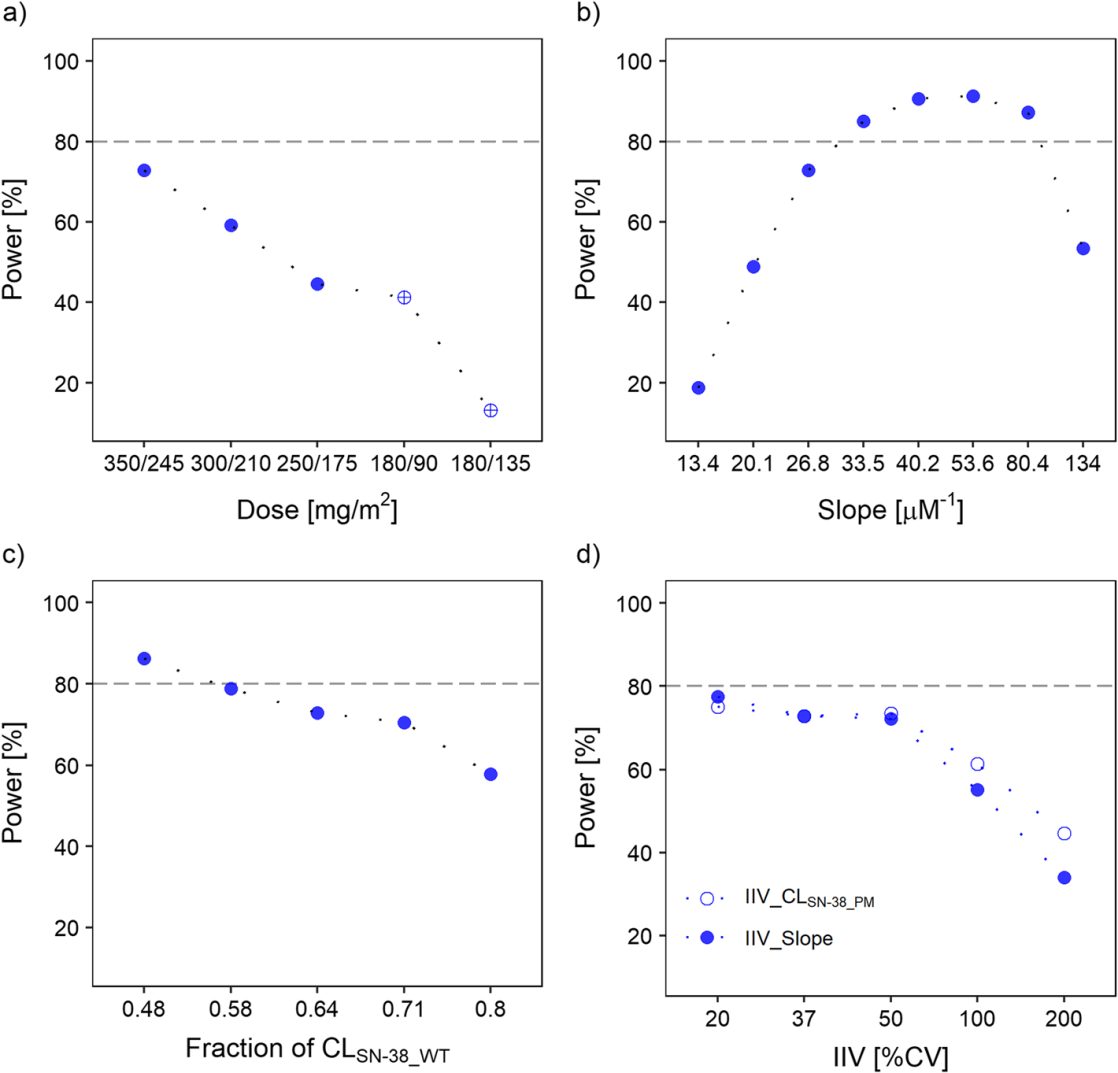
Power of different study designs given a) varied doses of irinotecan (standard dose/PGx-adjusted dose for poor metabolizers), b) perturbed values of the parameter *slope*, c) varied magnitudes of the parameter *CL*_*SN-38_PM*_ (PGx effect, expressed as fraction of *CL*_*SN-38_WT*_ = 1040 L/h) and d) different unexplained inter-individual variability associated with *slope* and *CL*_*SN-38_PM*_. Scenarios depicted in the plots were based on a parallel design with sample size *n* = 200/study arm. In Fig. 5a, filled circles represent irinotecan regimens with 30% difference between standard and PGx-based doses and empty circles depict lower irinotecan doses as used in combination chemotherapy regimens as well as dose reductions of −50% and − 25% as recommended for poor metabolizers. *CL*_*SN-38_WT*_: clearance of SN-38 in patients with wild-type UGT1A1*1/*1 genotype; *CL*_*SN-38_PM*_: clearance of SN-38 in poor metabolizers with respect to UGT1A1 (patients with *28/*28 or *1/*28 genotype); IIV: inter-individual variability; %CV: coefficient of variation.

Simulations of an approved *single-agent* regimen of irinotecan (350 mg/m^2^; default scenario) versus a 30% dose reduction in poor metabolizers (245 mg/m^2^) (corresponding to single doses of 345–600 mg) resulted in a study power of 73%. When choosing lower standard doses of 300 mg/m^2^ and 250 mg/m^2^, thereby keeping the 30% dose reduction for poor metabolizers, study power decreased to 59% and 45%, respectively (see Fig. 5a). The 180/90 mg/m^2^ regimens as used in *combination chemotherapy* regimens and entailing lower irinotecan doses and dose reductions beyond -30%, resulted in a study power of 41% similar to the abovementioned 250/175 mg/m^2^ regimens.

#### Drug-Related Model Parameters

Variation of the *slope* parameter, reflecting the potency of the exposure-response relationship in the neutropenia model, from 13.4 μM^−1^ up to 134 μM^−1^ (−50% to +400% of the original value) exerted a high influence on study power. The higher the assumed potency, the higher the corresponding power of the study was, but merely up to a peak (53.6 μM^−1^) from which the power decreased again (see Fig. 5b).

#### Patient-Related Model Parameters

Variation of the magnitude of the UGT1A1-related pharmacogenomic effect, i.e. the extent of CL_SN-38_ reduction in poor metabolizers, resulted in higher study power the lower CL_SN-38_ and consequently the higher the SN-38 exposure was (Fig. 5c).

#### Unexplained PK and PD Variability

A small impact of unexplained inter-individual variability on study power was revealed for low and moderate values of variability (≤50 %CV). High values of inter-individual variability, e.g. 200 %CV of the parameters *IIV_* CL_SN-38_PM_ and *IIV_Slope*, reduced study power to 45% and 34%, respectively (Fig. 5d).

### Evaluation of Study Designs by Model-Based Analysis

The model-based analysis approach suggested a statistically significant difference in the time course of neutrophils between the standard and PGx-based dosing regimens (350 mg/m^2^ versus 245 mg/m^2^). For both study designs, Model 1 (considering a difference in the neutrophil concentration-time profile after standard and PGx-based dosing) was statistically superior to Model 2 (mean ∆OFV: −32.5/−130 points for parallel/crossover design) for all model fits (*n* = 500), indicating a study power of 100%. The factor accounting for differences in the neutrophil concentration-time courses between standard and PGx-based dosing in the full model was estimated as 0.7 (median/minimum-maximum: parallel design: 0.74/0.62–0.86; crossover design: 0.72/0.65–0.79).

**Fig. 6:**
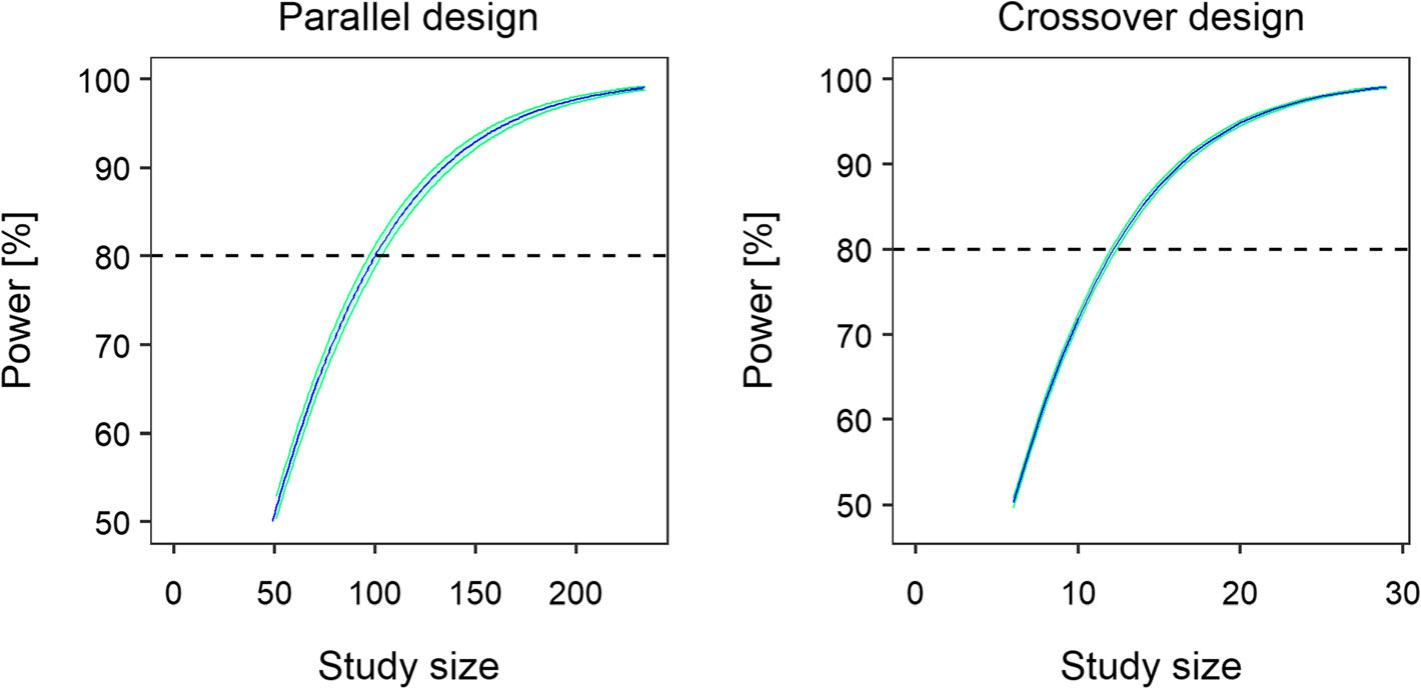
Full power versus sample size curves using parametric power estimation (PPE) based on SSE (stochastic simulation and estimation) for different study scenarios. Left panel: Parallel study design (see default study scenario); right panel: crossover study design (see alternative study scenario). The green shaded area indicates the uncertainty in the power estimate due to Monte-Carlo noise (*n* = 500). Power curves are shown for sample sizes corresponding to 50–99% power.

Figure 6 shows the power to detect significantly different hematological toxicity after PGx-based versus standard dosing for varied *sample sizes*, i.e. also beyond the originally simulated values. Given parallel study design, a total study size of *n* = 100 was found to result in 80% power and thus to be sufficiently large to identify statistically significantly lower hematological toxicity in patients receiving PGx-based dosing. Using a crossover study design, even a number of 15 patients seemed to suffice for the same purpose. Evidently, the results also emphasized that the difference in the selected doses of irinotecan (350 versus 245 mg/m^2^) were large enough to detect significantly reduced hematological toxicity with reasonable power.

## Discussion

Our investigation used irinotecan as an example drug to illustrate the usage of population PK/PD models to inform the design of clinical pharmacogenetic trials. Previously developed PK and PD models were combined to predict the impact of UGT1A1 genotypes on irinotecan-induced myelosuppression. The parameters of the updated *PK model* appeared plausible, with CL_SN-38_ estimated as 1040 L/h in patients with *1/*1 genotype and as 669 L/h (−35.7%) in poor metabolizers (assumed as 62% of the population). The values thus encircled the estimate of CL_SN-38_ in the original model not considering pharmacogenetic information (712 L/h). The same trend was observed for Q_SN-38_, which was correlated with CL_SN-38_ (1290 L/h and 1690 L/h versus 1530 L/h) (20).

In the updated *myelosuppression model*, the system-related parameters were of the same magnitude as had been reported for other chemotherapeutic drugs and types of blood cells (mean transit time: 92.8 h versus 88.7–135 h; feedback parameter: 0.165 versus 0.132–0.230) (22, 29). The estimated neutrophil baseline (5.58·10^9^ cells/L)—as the third system-related parameter—plausibly reflected the median observed baseline neutrophil count (5.2·10^9^ cells/L). The drug-specific *slope* parameter quantifying the inhibitory effect of SN-38 on the proliferation of progenitor blood cells (26.8 μM^−1^) was higher than when based on CPT-11 (1.29 μM^−1^), which is in agreement with *in vitro* studies showing a considerably higher cytotoxic effect of SN-38 (30). Neutropenia was assessed for the first cycle of therapy supported by a previous investigation indicating neutropenic effects in *28/*28 patients already in the first cycle of treatment (35).

Corresponding to previous studies, *simulations* with the PK/PD model indicated a dose-dependent risk of severe neutropenia following irinotecan therapy, which was considerably increased in patients with reduced UGT1A1 activity (grade 4 neutropenia in wild-type/poor metabolizers: 14%/21%) (18, 24, 31). The values are comparable with previous evidence, although results of former studies were variable. For example, Innocenti et al. found 50% grade 4 neutropenia in patients homozygous for the UGT1A1*28 allele, 12% in patients heterozygous for the *28 allele and 0% in patients with UGT*1/*1 (19). In line with the current analysis, other studies did find severe hematological toxicity also in wild-type patients (31). Chiara et al. assessed reduced doses of mono-agent therapy (180 mg/m^2^) and revealed a higher incidence of hematological toxicity (≥24%) than our analysis (10.7% following 245 mg/m^2^), though jointly included grade 3 and 4 neutropenia (18).

Pharmacometrics-based clinical trial simulations have proven useful to *a priori* evaluate the design of future studies and their feasibility in different therapeutic fields and drug development phases, particularly in case of challenging patient populations (e.g. pediatric patients) and practical constraints, e.g. with respect to enrolment numbers (e.g. phase I studies) (9–11, 32). Dickinson et al. demonstrated the utility of virtual pharmacogenetic trials using the example of warfarin and CYP2C9 polymorphisms, though without power analyses (33). Our evaluation revealed the consequence of different study design features, drug- and patient-related characteristics, model parameters as well as data analysis methods on study power, i.e. the detectability of a significant difference in the occurrence of grade 4 neutropenia after PGx-based dosing of irinotecan. Both *study design* (parallel/crossover) and the* magnitude of the PGx effect* (i.e. CL_SN-38_ reduction in poor metabolizers) exerted a high impact on study power. The investigated crossover design appeared as the most optimistic scenario. A lower required sample size was expected, as comparisons between different treatments are performed within the same patients and within-subject variability is typically lower than between-subject variability.

A higher *potency* of the drug-effect relationship (represented by the *slope* parameter) influenced the study power positively up to a limit, from which power decreased. The finding is plausible given that at high potency, proportions of grade 4 neutropenia are high both after standard and PGx-based dosing and only marginally different. In contrast, a difference in grade 4 neutropenia after standard versus PGx-based dosing is distinguishable when the potency of the drug is low. As for irinotecan *dosing*, the power to detect a difference in severe neutropenia was similar for treatment arms with 250 mg/m^2^ versus 175 mg/m^2^ and 180 mg/m^2^ versus 90 mg/m^2^. In the latter case, the lower absolute dose levels were compensated by a higher deviation between standard and PGx-based dosing. *Inter-individual variability* on the parameters slope and CL_SN-38_ propagates to variability in neutropenia and decreased study power merely for high magnitudes >50 CV%, which are still realistic as had been shown for other myelosuppressive agents (22).

The current study showed an advantage of model-based analysis of clinical trial data towards higher statistical power and considerably smaller sample sizes compared to traditional group-wise statistical analysis. In line with our finding, higher efficiency of model-based analysis (resulting in ≤8.5 times lower sample sizes) has been demonstrated in diverse studies of other therapeutic fields, e.g. in the area of stroke, diabetes, hyperhidrosis and tuberculosis (8, 34–36). The higher information content of model-based analyses emerges from the consideration of all available longitudinal information (e.g. the full time course of observations) rather than just a snapshot (e.g. last study observation) or summary measure (e.g. AUC) (37). The current study provides a novel example of model-based analysis, focusing on the implications of PGx for dosage adjustments. Further applications of the approach are conceivable for other settings, e.g. to investigate covariates or biomarkers that may drive the PK, effect and dosing.

The SSE revealed a typical estimate of the factor accounting for differences in myelosuppression after standard versus PGx-based dosing lower than 1 (0.74/0.72 for the parallel/crossover design), implying that administered doses lower than the standard (252/259 mg/m^2^) provoked the neutropenia. The estimated parameters were in good agreement with the originally simulated PGx-adjusted dose (245 mg/m^2^, representing a fraction of 0.7 of the standard dose), and in a wider sense indicated that hematological toxicity was significantly lower in patients receiving adjusted PGx-based dosing than in patients receiving a standard dose of irinotecan.

The main objective and strength of the conducted analysis was to illustrate the concept and opportunities of model-based trial design rather than to plan a concrete study. If the clinical feasibility of the investigated study designs was to be judged, the frequency of relevant PGx variants would require consideration. The presence of UGT1A1*28/*28 has been reported as ~12% in Caucasians, inferring that ~8 times higher patient numbers than identified in the above-described analyses would be necessary for the screening phase of the study or more when addressing drop-outs (21). For a parallel study with 80% power analyzed using standard statistical analysis, ~3700 patients (220 per treatment group·2/0.12) would have to be screened to enable to detect a significant difference in severe neutropenia between the two treatment groups. The necessitated sample size of this worst-case scenario seems large–possibly clinically unfeasibly large. On the other hand, a study with crossover design analyzed using the presented model-based approach would require merely 125 patients (15/0.12), suggesting a clinically possible alternative. While crossover studies require smaller sample sizes to obtain the same statistical power as parallel designs, however, a carry-over effect between the treatment periods might pose a hurdle and a likely increased risk of attrition owing to longer study durations might require consideration. Yet, our exemplary analysis illustrates how pharmacometric PK/PD models, i.e. the simulation and evaluation of reasonable study conditions (e.g. different study design-, drug- and patient-specific features) as well as data analysis methods and their consequences on study power, can help to avoid unfeasible, inconclusive trials and aid and rationalize the design of pharmacogenomics studies.

Some limitations should be acknowledged with regard to the present investigation. First, the results of clinical trial simulations are naturally depending on the assumptions of the underlying model. The original model quantifying the impact of UGT1A1 polymorphisms on the pharmacokinetics of irinotecan identified a 35.7% reduction of CL_SN-38_ jointly for patients homozygous and heterozygous for the *28 allele (i.e. genotypes *28/*28 and *1/*28) (17). Valenzuela et al. had additionally estimated a CL_SN-38_ reduction solely for patients carrying the *28/*28 genotype (ΔCL_SN-38_ 40.0%), though based on a data basis of only four patients, which was not deemed solid. Thus, the presented results might slightly underestimate SN-38 exposure and consequently the associated neutropenic effect in poor metabolizers with homozygous status of UGT1A1*28. Besides, further genetic variants of UGT1A1 (e.g. UGT1A1*93) and other enzymes and transporters (e.g. ABCC1, ABCB1) have been associated with irinotecan metabolism and might be additionally worth investigating (38, 39). Next, the employed PK/PD model did not consider inter-occasion variability (IOV), accounting for random deviations in the parameter estimates of an individual between different occasions. High IOV has been shown to limit the predictability from one treatment sequence to the next in a crossover design (40). However, a previous study has alleviated these concerns using the same model of chemotherapy-induced neutropenia as in the present analysis. Hansson et al. showed a clearly lower impact of IOV (implemented on the parameters *baseline*, *mean transit time*, and *slope*) than IIV in myelosuppression parameters on the variability in neutrophil counts (nadir) across six anti-cancer drug treatments. Furthermore, no systematic shifts in the system- or drug sensitivity-related parameters were observed over time, suggesting that the model can be used for predictions of future neutrophil-time courses, e.g. in a crossover study (41).

It is important to bear in mind that the myelosuppression model used for simulations was originally developed for single-agent irinotecan therapy; thus, the reported frequencies of grade 4 neutropenia may illustrate a ‘best-case scenario’ and not entirely mimic those after combination chemotherapy. Last, the parameter estimates of the model and study results are based on Caucasians and might not be directly transferable to other populations, not least as the prevalence of the *28 allele differs between ethnicities (21).

## Conclusion

In summary, we present a showcase project using irinotecan to illustrate how pharmacometric PK/PD models may serve as valuable tools to aid the design of clinical pharmacogenomics studies. A population PK/PD model accommodating PGx information was created to predict the occurrence of myelosuppression in cancer patients carrying different genotypes of UGT1A1 following different dosing strategies of irinotecan. The simulation and evaluation of reasonable study conditions and consequences of different study design, drug and patient features on study power together with the application of model-based analysis can help to avoid unfeasible, inconclusive studies and ultimately rationalize the design of pharmacogenetic studies.

### ACKNOWLEDGMENTS AND DISCLOSURES

The research leading to these results has received funding from the European Community’s Horizon 2020 Programme under grant agreement No. 668353 (U-PGx). The irinotecan PK and PD data were made available to us by Dr. Mark Ratain.

## Supplementary Information

ESM 1 (PDF 311 kb)

## Abbreviations

AUC: Area under the concentration-time curve
BSA: Body surface area
CI: Confidence interval
CPT-11: Irinotecan
CV: Coefficient of variation
DPWG: Dutch Pharmacogenetics Working Group
IIV: Inter-individual variability
IOV: Interoccasion variability
OFV: Objective function value
PD: Pharmacodynamic
PGx: Pharmacogenomics
PK: Pharmacokinetic
PM: Poor metabolizers
SN-38: 7-ethyl-10-hydroxycamptothecin
SN-38-G: SN-38-glucuronide
SSE: Stochastic simulation and estimation
UGT1A1: Uridine diphosphate-glucuronosyltransferase 1A1
VPC: Visual predictive check
WT: Wild-type

## References

[1] Lauschke VM, Milani L, Ingelman-Sundberg M. Pharmacogenomic Biomarkers for Improved Drug Therapy—Recent Progress and Future Developments. AAPS J. 2017;20(1). 10.1208/s12248-017-0161-x 29181807

[2] Relling MV, Klein TE, Gammal RS, Whirl-Carrillo M, Hoffman JM, Caudle KE. The clinical pharmacogenetics implementation consortium: 10 years later. Clin Pharmacol Ther. 2020;107(1):171–5. 10.1002/cpt.1651 PMC6925644 31562822

[3] Swen JJ, Nijenhuis M, De Boer A, Grandia L (2011). Maitland-van Der zee AH, Mulder H, et al. Pharmacogenetics: from bench to byte—an update of guidelines. Clin Pharmacol Ther.

[4] Bank PCD, Caudle KE, Swen JJ, Gammal RS, Whirl-Carrillo M, Klein TE, et al. Comparison of the guidelines of the Clinical Pharmacogenetics Implementation Consortium and the Dutch Pharmacogenetics Working Group. Clin Pharmacol Ther. 2018;103(4):599–618. 10.1002/cpt.762 PMC5723247 28994452

[5] Chenoweth MJ, Giacomini KM, Pirmohamed M, Hill SL, van Schaik RHN, Schwab M (2020). Global pharmacogenomics within precision medicine: challenges and opportunities. Clin Pharmacol Ther.

[6] Marshall SF, Burghaus R, Cosson V, Cheung SYA, Chenel M, DellaPasqua O (2016). Good practices in model-informed drug discovery and development: practice, application, and documentation. CPT Pharmacometrics Syst Pharmacol.

[7] Darwich AS, Ogungbenro K, Vinks AA, Powell JR, Reny JL, Marsousi N, et al. Why has model-informed precision dosing not yet become common clinical reality? Lessons from the past and a roadmap for the future. Clin Pharmacol Ther. 2017;101(5):646–56. 10.1002/cpt.659 28182269

[8] Karlsson KE, Vong C, Bergstrand M, Jonsson EN, Karlsson MO (2013). Comparisons of analysis methods for proof-of-concept trials. CPT Pharmacometrics Syst Pharmacol.

[9] Van Hasselt JGC, Eijkelenburg NKA, Beijinen JH, Schellens KHM, Huitema ADR. Design of a drug-drug interaction study of vincristine with azole antifungals in pediatric cancer patients using clinical trial simulation. Pediatr Blood Cancer. 2008;50(5):1018–25. 10.1002/pbc.25198 25175364

[10] Langenhorst JB, Dorlo TPC, van Kesteren C, van Maarseveen EM, Nierkens S, de Witte MA, et al. Clinical trial simulation to optimize trial design for fludarabine dosing strategies in allogeneic hematopoietic cell transplantation. CPT Pharmacometrics Syst Pharmacol. 2020;9(5):272–81. 10.1002/psp4.12486 PMC7239337 31957334

[11] Ibrahim MMA, Ghadzi SMS, Kjellsson MC, Karlsson MO (2018). Study design selection in early clinical anti-hyperglycemic drug development: a simulation study of glucose tolerance tests. CPT Pharmacometrics Syst Pharmacol.

[12] Deyme L, Barbolosi D, Gattacceca F (2019). Population pharmacokinetics of FOLFIRINOX: a review of studies and parameters. Cancer Chemother Pharmacol.

[13] Pfizer. Camptosar® (Irinotecan) Injection, intravenous infusion - Prescribing information. 2019. Available at: https://www.accessdata.fda.gov/drugsatfda_docs/label/2019/020571s050lbl.pdf.

[14] Dean L, Pratt V, McLeod H, Rubinstein W, Dean L, Kattman B, Malheiro A (2012). Irinotecan Therapy and UGT1A1 Genotype. Medical Genetics Summaries.

[15] Pommier Y, Pourquier P, Fan Y, Strumberg D (1998). Mechanism of action of eukaryotic topoisomerase II and drugs targeted to the enzyme. Biochim Biophys Acta.

[16] Barbarino JM, Haidar CE, Klein TE, Altman RB (2014). PharmGKB summary: very important pharmacogene information for UGT1A1. Pharmacogenet Genomics.

[17] Valenzuela Jiménez B, González Sales M, Escudero Ortiz V, Martínez Navarro E, Pérez Ruixo C, Rebollo Liceaga J, et al. Influencia de los polimorfismos genéticos en UGT1A1, UGT1A7 y UGT1A9 sobre la farmacocinética de irinotecán, SN-38 y SN-38G. Farm Hosp. 2013;37(2):111–27. 10.7399/FH.2013.37.2.386 23789755

[18] Hoskins JM, Goldberg RM, Qu P, Ibrahim JG, McLeod HL (2007). UGT1A1*28 genotype and irinotecan-induced neutropenia: dose matters. J Natl Cancer Inst.

[19] Innocenti F, Undevia SD, Iyer L, Chen PX, Das S, Kocherginsky M (2004). Genetic variants in the UDP-glucuronosyltransferase 1A1 gene predict the risk of severe neutropenia of irinotecan. J Clin Oncol.

[20] Xie R, Mathijssen RHJ, Sparreboom A, Verweij J, Karlsson MO (2002). Clinical pharmacokinetics of irinotecan and its metabolites in relation with diarrhea. Clin Pharmacol Ther.

[21] KNMP. General background text Pharmacogenetics - UGT1A1. 2014; Available from: https://www.knmp.nl/downloads/g-standaard/farmacogenetica/english-background-information/ugt1a1-english.pdf

[22] Friberg LE, Henningsson A, Maas H, Nguyen L, Karlsson MO (2002). Model of chemotherapy-induced myelosuppression with parameter consistency across drugs. J Clin Oncol.

[23] Sacco JJ, Botten J, Macbeth F, Bagust A, Clark P (2010). The average body surface area of adult cancer patients in the UK: a multicentre retrospective study. PLoS One.

[24] Sharma MR, Joshi SS, Karrison TG, Allen K, Suh G, Marsh R, et al. A UGT1A1 genotype-guided dosing study of modified FOLFIRINOX in previously untreated patients with advanced gastrointestinal malignancies. Cancer. 2019;125(10):1629–36. 10.1002/cncr.31938 30645764

[25] U.S. Department of Health and Human Services. Common Terminology Criteria for Adverse Events (CTCAE) v5.0. 2017; Available from: https://ctep.cancer.gov/protocolDevelopment/electronic_applications/ctc.htm

[26] Keizer RJ, Karlsson MO, Hooker A (2013). Modeling and simulation workbench for NONMEM: tutorial on Pirana, PsN, and Xpose. CPT Pharmacometrics Syst Pharmacol.

[27] Ueckert S (2016). Accelerating Monte Carlo power studies through parametric power estimation. J Pharmacokinet Pharmacodyn.

[28] Beal SL, Sheiner LB, Boeckmann AJ, Bauer RJ. (Eds). NONMEM 7.4 users guides (1989–2018). https://nonmem.iconplc.com/nonmem743/guides.

[29] Henrich A, Joerger M, Kraff S, Jaehde U, Huisinga W, Kloft C, et al. Semimechanistic bone marrow exhaustion pharmacokinetic/pharmacodynamic model for chemotherapy-induced cumulative neutropenia. J Pharmacol Exp Ther. 2017;362(2):347–58. 10.1124/jpet.117.240309 28600397

[30] Kawato Y, Aonuma M, Hirota Y, Kuga H, Sato K. Intracellular roles of SN-38, a metabolite of the camptothecin derivative CPT-11, in the antitumor effect of CPT-11. Cancer Res. 1991;51(16):4187–91. 1651156

[31] Côté JF, Kirzin S, Kramar A, Mosnier JF, Diebold MD, Soubeyran I, et al. UGT1A1 polymorphism can predict hematologic toxicity in patients treated with irinotecan. Clin Cancer Res. 2007;13(11):3269–75. 10.1158/1078-0432.CCR-06-2290 17510208

[32] Ueckert S, Hennig S, Nyberg J, Karlsson MO, Hooker AC (2013). Optimizing disease progression study designs for drug effect discrimination. J Pharmacokinet Pharmacodyn.

[33] Dickinson GL, Lennard MS, Tucker GT, Rostami-Hodjegan A (2007). The use of mechanistic DM-PK-PD modelling to assess the power of pharmacogenetic studies - CYP2C9 and warfarin as an example. Br J Clin Pharmacol.

[34] Mehrotra S, Schmith VD, Dumitrescu TP, Gobburu J (2015). Pharmacometrics-guided drug development of antihyperhidrosis agents. J Clin Pharmacol.

[35] Svensson RJ, Gillespie SH, Simonsson USH (2017). Improved power for TB phase IIa trials using a model-based pharmacokinetic-pharmacodynamic approach compared with commonly used analysis methods. J Antimicrob Chemother.

[36] Karlsson KE, Grahnén A, Karlsson MO, Jonsson EN (2007). Randomized exposure-controlled trials; impact of randomization and analysis strategies. Br J Clin Pharmacol.

[37] Tessier A, Bertrand J, Chenel M, Comets E. Comparison of nonlinear mixed effects models and noncompartmental approaches in detecting pharmacogenetic covariates. AAPS J. 2015;17(3):597–608. 10.1208/s12248-015-9726-8 PMC4406951 25693489

[38] Innocenti F, Kroetz DL, Schuetz E, Dolan ME, Ramírez J, Relling M, et al. Comprehensive pharmacogenetic analysis of irinotecan neutropenia and pharmacokinetics. J Clin Oncol. 2009;27(16):2604–14. 10.1200/JCO.2008.20.6300 PMC2690389 19349540

[39] Li M, Seiser EL, Baldwin RM, Ramirez J, Ratain MJ, Innocenti F, et al. ABC transporter polymorphisms are associated with irinotecan pharmacokinetics and neutropenia. Pharmacogenomics J. 2018;18(1):35–42. 10.1038/tpj.2016.75 PMC5432414 27845419

[40] Wallin JE, Friberg LE, Karlsson MO (2009). Model-based neutrophil-guided dose adaptation in chemotherapy: evaluation of predicted outcome with different types and amounts of information. Basic Clin Pharmacol Toxicol.

[41] Hansson EK, Wallin JE, Lindman H, Sandström M, Karlsson M, Friberg L (2010). Limited inter-occasion variability in relation to inter-individual variability in chemotherapy-induced myelosuppression. Cancer Chemother Pharmacol.

