# Supplementary material for: Pharmacometrics-Based Considerations for the Design of a Pharmacogenomic Clinical Trial Assessing Irinotecan Safety
Source: Pharm Res. 2021 Mar 17;38(4):593–605. doi: 10.1007/s11095-021-03024-w (PMC8057977; doi:10.1007/s11095-021-03024-w)
Supplement: Supplementary file 1 — (PDF 311 kb) [file 11095_2021_3024_MOESM1_ESM.pdf]

Electronic Supplementary Material

Pharmaceutical Research

Pharmacometrics-based considerations for the design of a pharmacogenomic clinical trial assessing irinotecan safety

Iris K. Minichmayr[1], Mats O. Karlsson[1], Siv Jönsson[1*]

[1]Department of Pharmacy, Uppsala University, Box 580, 75123 Uppsala, Sweden

*Corresponding author. Mailing address: Box 580, 75123 Uppsala, Sweden;
Tel: +46 733 924 657; Fax: -
E-mail: siv.jonsson@farmaci.uu.se

Preliminary determination of the sample size for a study with parallel design

The required sample size for each arm of a two-treatment parallel study was calculated analytically, without the involvement of a PK/PD model, based on a statistical test (chi-squared test) comparing two pre-specified proportions (e.g. proportions of patients with grade 4 neutropenia after standard versus PGx-based dosing), a defined significance level ($\alpha=0.05$) and a desired study power (80%). The specified difference in proportions corresponded to the desired reduction in the endpoint 'grade 4 neutropenia' that a study shall detect when administering PGx-based instead of standard doses. A previous study has demonstrated a prevalence of grade 4 neutropenia of 50% in patients with *28/*28 genotype following the same standard dose as used in the study design evaluation (350 mg/m^2 as a 90-minute infusion, every 3 weeks) (20). Further evidence has also suggested lower rates of severe neutropenia (e.g. 36% and lower) (14,47). When assuming 50% or 35% patients with grade 4 neutropenia based on this prior knowledge, 170 or 300 patients would be necessary per study arm to detect a significant difference in proportions of 30%, as targeted in a vast multi-center study on preemptive pharmacogenomic testing for irinotecan and other agents in Europe (48). Based on the preliminary exploration, an exemplary sample size of 200 patients per treatment arm was chosen as a starting point for further investigations of different study designs and exploration of clinically reasonable study conditions.

Supplementary Figures

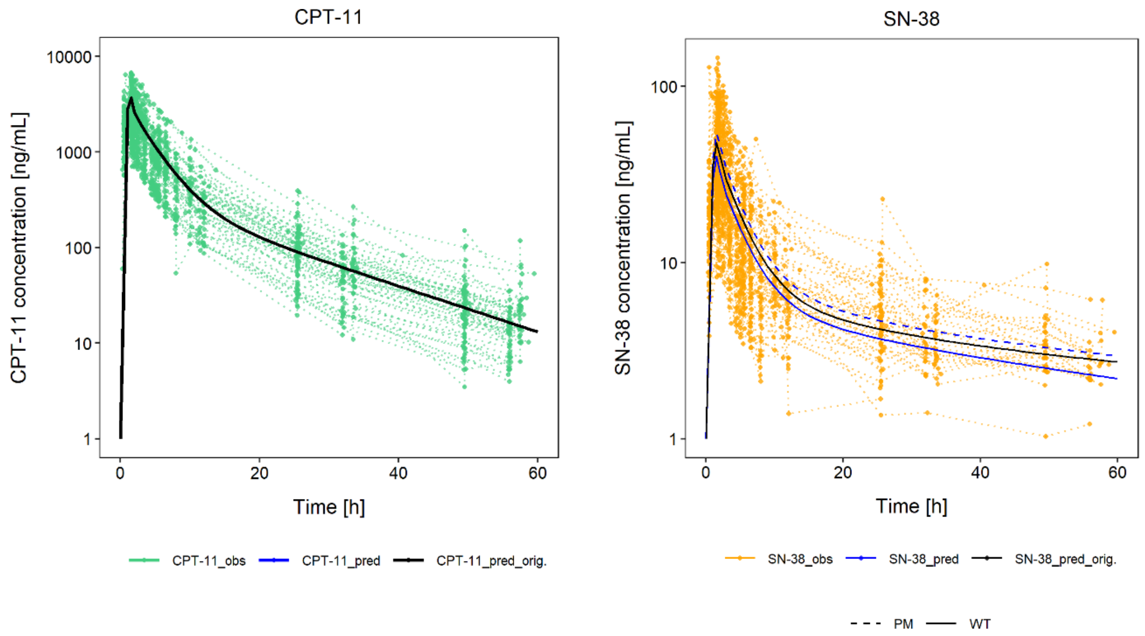

Figure S1. Observed concentration-time profiles of CPT-11 and SN-38 (colored dotted lines) and model-predicted typical profiles based on the original model without PGx information (black solid line; Xie et al. (23)) versus the updated model, including PGx information (UGT1A1-based clearance reduction of SN-38, blue lines). Blue solid lines indicate concentration-time profiles of metabolites in patients with wild-type (WT) genotype (*1/*1); dashed blue lines represent concentration-time profiles in poor metabolizers (PM, genotypes *28/*28 and *1/*28). Simulations of the typical pharmacokinetic profiles were based on a dose of 300 mg/m^2 (in proximity to the median dose underlying the original model). Irinotecan doses administered in the patient population underlying the original model ranged from 100-350 mg/m^2, causing diverse concentration-time courses of irinotecan and its metabolites. The shown time span of 60 h corresponds to the observation period during which concentrations of the different entities had been measured. Body surface area corresponded to the median in the population (1.87 m^2).

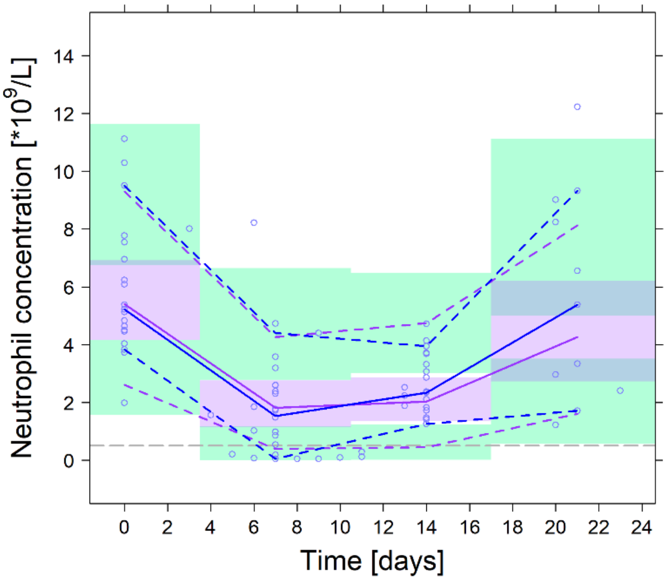

Figure S2. Visual predictive check for the observed neutrophil concentrations (open circles) during irinotecan therapy. The blue solid line depicts the median of the observations; the purple solid line represents the median of the model predictions. The dashed lines represent the 10th and 90th percentiles of the observed (blue) and predicted (purple) data. The shaded areas represent the 95% confidence intervals for the predicted percentiles. The grey dashed horizontal line indicates grade 4 neutropenia ($0.5 \cdot 10^9$ cells/L).
